# Acute reverse annular remodeling during MitraClip^®^ therapy predicts improved clinical outcome in heart failure patients: a 3D echocardiography study

**DOI:** 10.1186/s40001-017-0273-x

**Published:** 2017-09-20

**Authors:** Theresa Herbrand, Silke Eschenhagen, Tobias Zeus, Eva Kehmeier, Katharina Hellhammer, Verena Veulemans, Malte Kelm, Jan Balzer

**Affiliations:** Department of Medicine, Division of Cardiology, Pneumology, and Angiology, University Hospital Duesseldorf, Moorenstr. 5, 40225 Duesseldorf, Germany

**Keywords:** TMVR, Mitral valve insufficiency, Three-dimensional, Echocardiography, Remodeling, MitraClip^®^

## Abstract

**Background:**

Transcatheter mitral valve repair (TMVR) has been shown to have acute effects on mitral valve geometry in patients with functional mitral regurgitation (FMR). This study investigates the impact of MitraClip^®^ therapy-induced annular remodeling on clinical outcome and mitral regurgitation in heart failure patients.

**Methods:**

TMVR was performed successfully in 45 patients with FMR. In this study, mitral valve datasets were obtained before and directly after MitraClip^®^ implantation using three-dimensional (3D) transesophageal echocardiography, and were analyzed offline retrospectively using dedicated 3D reconstruction software. Patients underwent clinical and echocardiographic evaluation at baseline and after 6 months. At follow-up, the patients were allocated into two groups according to their improvement in New York Heart Association (NYHA) functional class: a Low Responder group with ΔNYHA <1.5 (*n* = 25); and a High Responder group with ΔNYHA ≥1.5 (*n* = 20).

**Results:**

At 6-month follow-up, data analysis revealed that while mitral regurgitation was reduced significantly in both groups, only the High Responder group had experienced significant downsizing of the 3D circumference (137 ± 14 mm to 126 ± 13 mm; *p* < 0.01) and the anterior-to-posterior diameter (33 ± 5 mm to 29 ± 4 mm; *p* < 0.01) of the mitral annulus during the intervention. Furthermore, only the High Responder group with reverse annular remodeling as shown had substantial advances in quality of life (Minnesota living with heart failure questionnaire: 55 ± 10 to 34 ± 14 points; *p* < 0.01) and functional status (6-min walk distance: 290 ± 104 m to 462 ± 111 m; *p* = 0.07).

**Conclusion:**

Our study demonstrates that instantaneous left ventricular annular remodeling during MitraClip^®^ implantation is associated with improved clinical outcome of heart failure patients with functional mitral regurgitation.

*Trial registration* The study was approved by the local ethics committee (Study Number 4497R, Registration ID: 2013121585). Trial registration: NCT02033811 Retrospectively registered January 9, 2014.

## Background

Heart failure patients are characterized by severe left ventricular dysfunction with intact valve leaflets leading to functional mitral regurgitation (FMR), which is associated with a poor overall prognosis [1]. Transcatheter mitral valve repair (TMVR) using the MitraClip^®^ system has become an additional, low-risk, successful treatment option [2, 3], even for multimorbid patients suffering from diabetes, renal dysfunction, or anemia [4–6]. TMVR reduces the severity of mitral regurgitation (MR) and leads to reversed cardiac remodeling, increased ejection fraction, and improved functional status over time [7]. Furthermore, the degree of left ventricular reverse remodeling in patients with FMR is directly associated with the immediate magnitude of reduction in regurgitation severity during the procedure [8]. Therefore, in contrast to patients with degenerative mitral regurgitation (DMR), in patients with FMR, TMVR has acute intra-procedural effects on mitral ring geometry, as demonstrated by a three-dimensional (3D) transesophageal echocardiography (TEE) study [9]. Most importantly, there is evidence from a recent study suggesting that clinical response to TMVR in patients with FMR is not only influenced by the immediate reduction of mitral regurgitation severity alone, but also by effects of the MitraClip^®^ on mitral valve geometry leading to acute downsizing of the mitral annulus during the procedure [10]. This observation is supported by the results of the recent TITAN II trial which could demonstrate that percutaneous mitral annuloplasty using the carillon device leads to clinical improvement in patients with FMR without using an edge-to-edge technology [11]. However, it is yet unclear whether the reduction of mitral regurgitation or the remodeling effect of the MitraClip^®^ on the mitral valve annulus is the stronger predictor of clinical outcome in patients with heart failure and consecutive FMR. We therefore aimed to investigate whether improvement of functional status is associated with reverse annular remodeling during TMVR in heart failure patients with FMR.

## Methods

### Patient characteristics

**Fig. 1:**
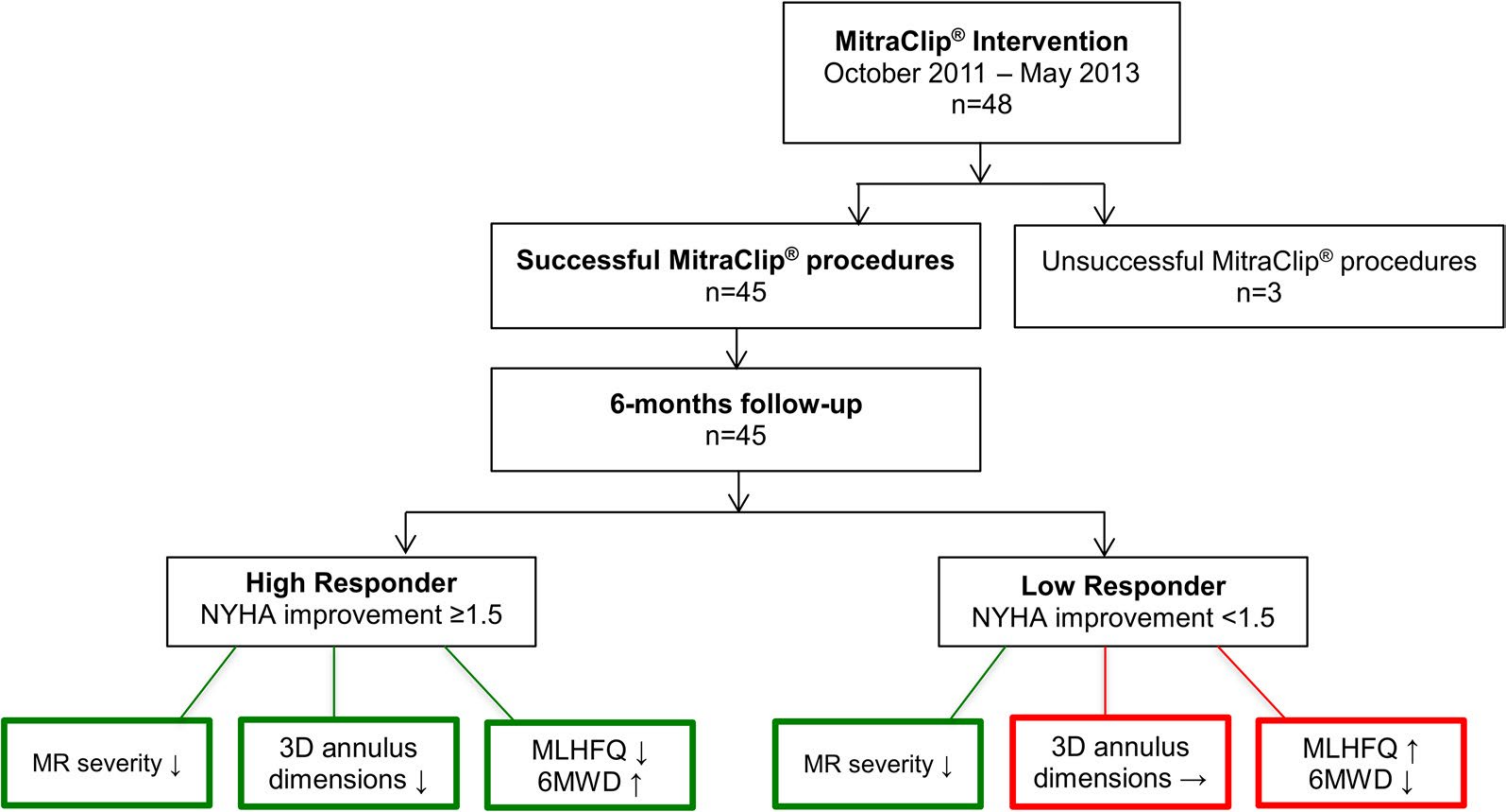
Flowchart of patients who underwent TMVR. Unsuccessful procedures were attributed to difficult anatomy in two cases and a thrombus in the left atrium in one case. One patient was previously excluded from the study despite successful TMVR due to participation in another trial. *MR* mitral regurgitation, *MLHFQ* Minnesota living with heart failure questionnaire, *MVQ* mitral valve quantification, *NYHA* New York Heart Association, *6MWD* 6-min walk distance

In this study, symptomatic heart failure patients with FMR considered inoperable according to a previous heart team decision, New York Heart Association (NYHA) class II–IV, and an ejection fraction <35% were accepted for TMVR in accordance with current guidelines [12]. Patients with primary degenerative mitral regurgitation (DMR) were excluded from the study. In total, 48 heart failure patients were enrolled (Fig. 1). In three patients, the MitraClip^®^ procedure was unsuccessful. In two of those 3 patients, the posterior leaflet could not be gripped by the device, and in one patient a thrombus was detected in the left atrial appendage at the beginning of the procedure so that TMVR was discontinued. Subsequently, 45 patients were included for final data analysis. The study was approved by the local ethics committee and all patients gave written informed consent before study inclusion.

### Transcatheter mitral valve repair (TMVR)

MitraClip^®^ implantation was performed as previously described [13]. The procedure was conducted under general anesthesia, and was performed by the same two experienced interventionists using fluoroscopy and one imaging cardiologist providing 2D and 3D TEE images.

### Image acquisition and follow-up investigations

Echocardiographic data were acquired during TMVR before and after MitraClip^®^ implantation using TEE. Follow-up after 6 months was performed using transthoracic echocardiography (TTE). Echocardiography was performed using a commercially available echocardiography system (iE 33, Philips Medical Systems, Andover, Massachusetts) with matrix array transducers (TEE: X7-2t; TTE: X5-1) capable of generating both two-dimensional (2D) and 3D images. Pre-procedural echocardiographic chamber quantification, left ventricular function assessment, and mitral regurgitation evaluation were performed according to current recommendations [14–16]. We graded the severity of MR as grade 1 (mild), grade 2 (moderate), grade 3 (moderate to severe), and grade 4 (severe), corresponding to the EVEREST criteria for quantification [13]. Echocardiographic image acquisition and presentation to the interventionist during the procedure was also performed according to current guidelines [17, 18]. Assessments such as 6-min walk distance and the Minnesota Living With Heart Failure Questionnaire were performed before and after TMVR. Additionally, N-terminal pro b-type natriuretic peptide (NT pro-BNP) levels were measured before TMVR and at follow-up.

### 3D analysis of mitral valvular geometry

Anatomical measurements were performed offline using dedicated software (MVQ QLAB version 8.1 Software 2010 (Philips, Andover, MA, USA)). After choosing an image at end-systole from 3D full-volume datasets and optimization in terms of size, contrast, and enhancement, the image was cropped to obtain a perfect en-face surgical view of the mitral valve. The cropped 3D image was then aligned along the transversal, horizontal, and sagittal planes by means of multiplanar reconstruction. The aligned image was used to mark reference points, such as the anterolateral, posteromedial, anterior, and posterior direction of the mitral valve annulus, and other anatomical landmarks, e.g., the aorta and the nadir of the mitral valve leaflets. Afterwards, semi-automated reconstruction was started, generating a virtual 3D model of the mitral valve apparatus. Adjustments of the mitral leaflet commissural points, the mitral leaflets, and the coaptation length were set. The coaptation points were set correctly with the help of the surgical view of the 3D image. The 3D model of the mitral valve now contained accurate measurements of the mitral valve geometry. All anatomical measurements were performed by the same well-trained, experienced investigator blinded to the numerical outcome of the measurements while adjusting.

### Definition of clinical response to TMVR

Patients were allocated by outcome into two groups, the High and Low Responders. High Responders (HR) were defined as patients with a decrease in NYHA classification ≥1.5 at 6 months after TMVR. Patients with a change in NYHA classification <1.5 were defined as Low Responders (LR), as only marginal clinical benefits were evident. This allocation was based on a recent echocardiographic study with a similar population [10].

### Statistical analysis

Normal distribution of continuous variables was examined using the D’Agostino-Pearson omnibus normality test. For normally distributed data (presented as mean ± SD), paired *t* tests were performed. For non-normally distributed data (presented as median and interquartile range), the Wilcoxon test was used. Comparisons of, e.g., mitral valve geometry before and after TMVR, were performed using *t* test or the Wilcoxon signed-rank test, depending on the distribution of data. Two-tailed *p* values <0.05 were considered to be significant; therefore, in case *p* value was equal or less than the chosen significance level, the null hypothesis had to be rejected. Categorical data were presented as frequencies and percentages. Graphics and statistical analysis were made using Excel for Mac 2011 (Version 14.1.0) and GraphPad Prism version 5.0b for MacOS X (GraphPad Software, San Diego, CA, USA).

## Results

### Clinical baseline characteristics and procedural outcome

In total, 45 patients (age 70 ± 11 years; 29 males) with FMR were successfully treated with TMVR, three of which received two clips. At baseline, 33 patients (73%) had severe (4+), 5 patients (11%) moderate to severe (3+), and 7 patients (16%) moderate (2+) MR. 34 patients (76%) had NYHA class III and higher. Baseline characteristics and interventional data are presented in Table 1. Atrial fibrillation was more common in the Low Responder group (84% vs. 45%; *p* < 0.005).

**Table 1:** Baseline characteristics

	All patients	High Responder	Low Responder	*p*
Age (yrs ± SD)	70 ± 11	67 ± 25	72 ± 11	0.15
Male* n* (%)	29 (64)	15 (75)	14 (56)	0.19
BMI (kg/m^2^ ± SD)	27 ± 5	27 ± 5	27 ± 5	0.69
Grade of MR* n* (%)				0.86
4+	33 (73)	14 (70)	19 (76)	
3+	5 (11)	4 (20)	1 (4)	
2+	7 (16)	2 (10)	5 (20)	
NYHA* n* (%)				0.06
IV	8 (18)	4 (20)	4 (16)	
III–IV	11 (24)	8 (40)	3 (12)	
III	15 (33)	5 (25)	10 (40)	
II–III	8 (18)	3 (15)	5 (20)	
II	3 (7)	0	3 (12)	
Previous interventions* n* (%)				
CABG or PCI	12 (27)	5 (25)	7 (28)	0.82
Valve surgery	4 (9)	1 (5)	3 (12)	0.42
ICD	13 (29)	8 (40)	5 (20)	0.15
CRT	9 (20)	3 (15)	6 (24)	0.46
Comorbidities* n* (%)				
AF	30 (67)	9 (45)	21 (84)	0.005
SR	21 (47)	12 (6)	9 (36)	0.11
CAD	29 (64)	11 (55)	18 (72)	0.25
Previous MI	15 (33)	5 25)	10 (40)	0.30
COPD	8 (18)	5 (25)	3 (12)	0.27
pHTN	29 (64)	14 (70)	15 (60)	0.51
Logistic EuroSCORE (% ± SD)	17 ± 17 (6–21)	16 ± 17	133 ± 17 (6–19)	0.84
Medication* n* (%)				
ACE inhibitors/ARB	29 (64)	13 (65)	16 (64)	0.95
AT1-antagonists	6 (13)	4 (20)	2 (8)	0.25
Beta-blockers	41 (91)	18 (90)	23 (92)	0.82
Loop diuretics	36 (80)	16 (80)	20 (80)	1
Aldosterone antagonists	22 (49)	12 (60)	10 (40)	0.19

*AF* atrial fibrillation, *BMI* Body Mass Index; *CABG*, coronary artery bypass graft, *CAD* coronar artery disease, *COPD* chronic obstructive pulmonary disease, *CRT* cardiac resynchronization therapy, *ICD* implantable cardioverter-defibrillator, *MI* myocardial infarction, *MR* mitral regurgitation, *NYHA* New York Heart Association, *PCI* percutaneous coronary intervention, *pHTN* pulmonary hypertension, *SR* sinus rhythm

### Echocardiographic parameters at baseline and outcome after 6-month follow-up

**Fig. 2:**
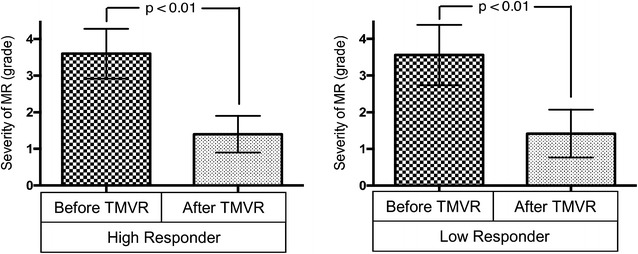
Severity of mitral regurgitation in High and Low Responders. *MR* mitral regurgitation, *TMVR* transcatheter mitral valve repair

In patients who underwent TMVR, overall, MR was reduced significantly (*p* < 0.01, Fig. 2). After the intervention, no patient had a significant increase of mean pressure gradient (i.e., increase of mean pressure gradient ≥5 mmHg) or pericardial effusion. 2D values such as vena contracta (VC) and systolic pulmonary artery pressure (PAsys) were examined. PAsys improved significantly before and after TMVR in the HR group compared to the LR group (meanΔ −6 ± 10 mmHg; *p* < 0.03 *vs*. meanΔ −1 ± 20; *p* = 0.8). Changes of echocardiographic parameters regarding mitral regurgitation were not significantly different between both groups. Furthermore, there were no significant changes between the groups and at follow-up for atrial and ventricular dimensions, left ventricular (LV), and right ventricular function. All echocardiographic parameters are summarized in Table 2.

**Table 2:** Echocardiographic and functional values at baseline and at 6-month follow-up

	Baseline			6-month follow-up		
	High Responder	Low Responder	*p*	High Responder	Low Responder	*p*
	*n* = 20	*n* = 25		*n* = 20	*n* = 25	
PISA (mm/s ± SD)	8 ± 2	8 ± 2	0.98	5 ± 1	5 ± 2	0.75
VC (mm ± SD)	6 ± 2	6 ± 2	0.59	4 ± 2	4 ± 1	0.79
PAsys (mmHg ± SD)	40 ± 14	36 ± 17	0.44	34 ± 11	35 ± 11	0.89
TAPSE (mm ± SD)	18 ± 3	19 ± 5	0.83	20 ± 6	19 ± 6	0.60
LA area (cm^2^ ± SD)	31 ± 9	29 ± 8	0.39	31 ± 7	29 ± 15	0.62
RA area (cm^2^ ± SD)	26 ± 6	25 ± 9	0.67	24 ± 9	23 ± 8	0.54
LVEDD (mm ± SD)	67 ± 10	57 ± 14	0.04	67 ± 11	61 ± 15	0.27
EF (% ± SD)	31 ± 8	32 ± 8	0.62	32 ± 8	37 ± 10	0.22
PE	0	0		0	0	
Grade of MR			0.86			0.92
4+	14	19		0	0	
3+	4	1		0	2	
2+	2	5		6	6	
1+	0	0		14	16	
NT pro-BNP (pg/mL ± SD)	2837 ± 1642	2070 ± 2605	0.43	1705 ± 1080	2782 ± 2632	0.22

*EF* ejection fraction, *LA* left atrial, *LVEDD* left ventricular end-diastolic diameter, *MR* mitral regurgitation, *NT pro*-*BNP* N-terminal pro b-type natriuretic peptide, *PAsys* systolic pulmonary artery pressure, *PE* pericardial effusion, *PISA* proximal isovelocity area, *RA* right atrial, *TAPSE* tricuspid annular plane systolic excursion, *VC* vena contracta

### Clinical response to TMVR after 6 months

**Fig. 3:**
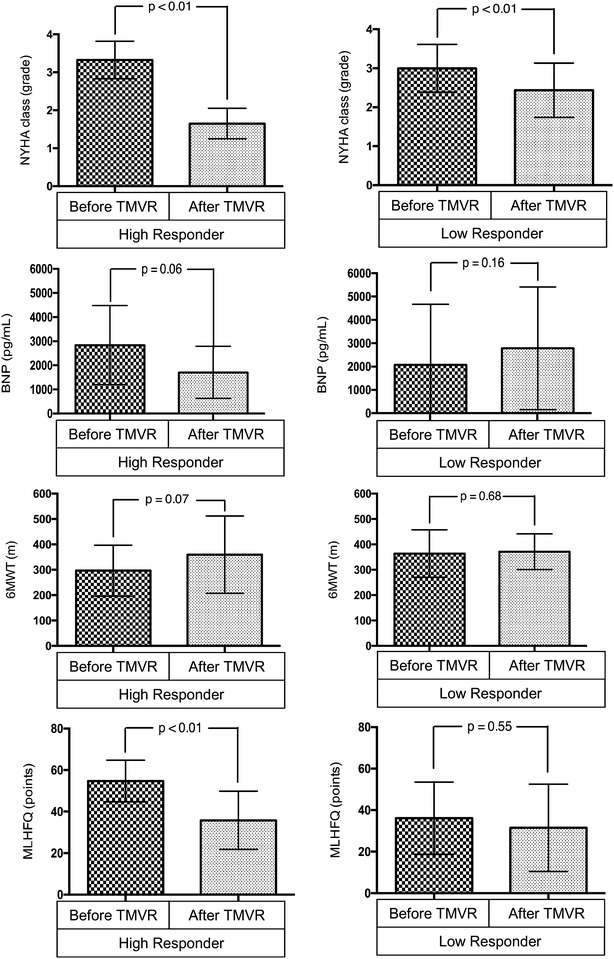
Statistical analysis. Clinical parameters are shown on the y-axis, pre- and postprocedural measurements on the x-axis, respectively. Each diagram contains the *p* value of measurements before and after TMVR. *MLHFQ* Minnesota Living With Heart Failure Questionnaire, *NT pro*-*BNP* N-terminal pro b-type natriuretic peptide, *NYHA* New York Heart Association, *6MWT* 6-min walk distance

NYHA class improvement after TMVR occurred in both groups, but was significantly higher in the HR group as compared to the LR group (HR: 3.5 ± 0.5 vs. 1.6 ± 0.5; *p* < 0.001; LR: 3 ± 0.6 vs. 2.5 ± 0.7; *p* < 0.01). Consistent with these results, the results of the Minnesota Living with Heart Failure Questionnaire (55 ± 10 points vs. 34 ± 14 points; *p* < 0.01) increased within the HR group with a similar trend in the 6-min walk distance (290 ± 104 m vs. 362 ± 110 m; *p* = 0.2), whereas these parameters barely changed in the LR group (6-min walk distance: 364 ± 94 points vs. 372 ± 70 points; *p* = 0.9; MLHFQ: 36 ± 17 vs. 32 ± 21; *p* = 0.7). NT pro-BNP decreased in the HR group (2837 ± 1642 pg/mL vs. 1705 ± 1080 pg/mL; *p* = 0.06). Data for clinical response of both groups after 6 months are presented in Fig. 3.

### Acute changes of 3D mitral valve geometry during the procedure

This study focused on the analysis of 3D values using QLAB. MVQ results before and immediately after TMVR for both groups are presented in Table 3. Results obtained from 3D data immediately before and after clip showed that anterior-to-posterior diameter (DAP) (meanΔ −4 ± 3.5; *p* < 0.01), 3D circumference (C3D) (meanΔ −11.5 ± 8.7; *p* < 0.01), and 2D circumference (C2D) (meanΔ −11.8 ± 10; *p* < 0.01) significantly decreased in HRs as shown in Table 3. The Low Responder group did not show any significant changes in C3D (*p* = 0.07) or DAP (*p* = 0.1). We did not find any significant changes in anterolateral to posteromedial diameters (DAlPm) in either group (*p* = 0.07 in High Responder group and *p* = 0.09 in Low Responder group).

**Table 3:** 3D Qlab values before and after TMVR in High Responders and Low Responders

Qlab values (mm ± SD)	High Responder			Low Responder		
	Before TMVR	After TMVR	*p*	Before TMVR	After TMVR	*p*
DAlPm	46 ± 6	45 ± 5	0.07	45 ± 11	44 ± 10	0.09
DAP	33 ± 5	29 ± 4	<0.01	32 ± 8	32 ± 8	0.1
C3D	137 ± 14	126 ± 13	<0.01	135 ± 33	133 ± 32	0.7
C2D	136 ± 14	124 ± 14	<0.01	121 ± 20	115 ± 16	<0.01

*C2D* 2D circumference, *C3D* 3D circumference, *DAlPm* anterolateral to posteromedial diameter, *DAP* anterior-to-posterior diameter

## Discussion

Our study demonstrates that instantaneous left ventricular annular remodeling during MitraClip^®^ implantation is associated with improved clinical outcome of heart failure patients with functional mitral regurgitation. These instantaneous alterations of mitral annulus geometry during the procedure can be delineated using 3D TEE and have a strong impact on functional status and quality of life. Our results underline the fact that the MitraClip^®^ system is an effective therapy for patients with heart failure at high surgical risk. Patients with highly increased clinical outcome and functional status showed, apart from the reduction of MR severity, TMVR-induced reversed remodeling of the mitral annulus, whereas patients without comparable clinical benefit only showed reduction of the MR jet.

In detail, the main findings of the present study are as follows:

1. TMVR has acute effects not only on MR severity but also on 3D mitral annulus geometry in heart failure patients with FMR.
2. Clinical response to TMVR in heart failure patients with FMR depends on the combination of 3D mitral annular remodeling and reduction of MR severity.
3. Reduction of mitral annular geometry using 3D TEE during TMVR is associated with improved clinical benefit.

### Acute effects of TMVR on mitral ring geometry

**Fig. 4:**
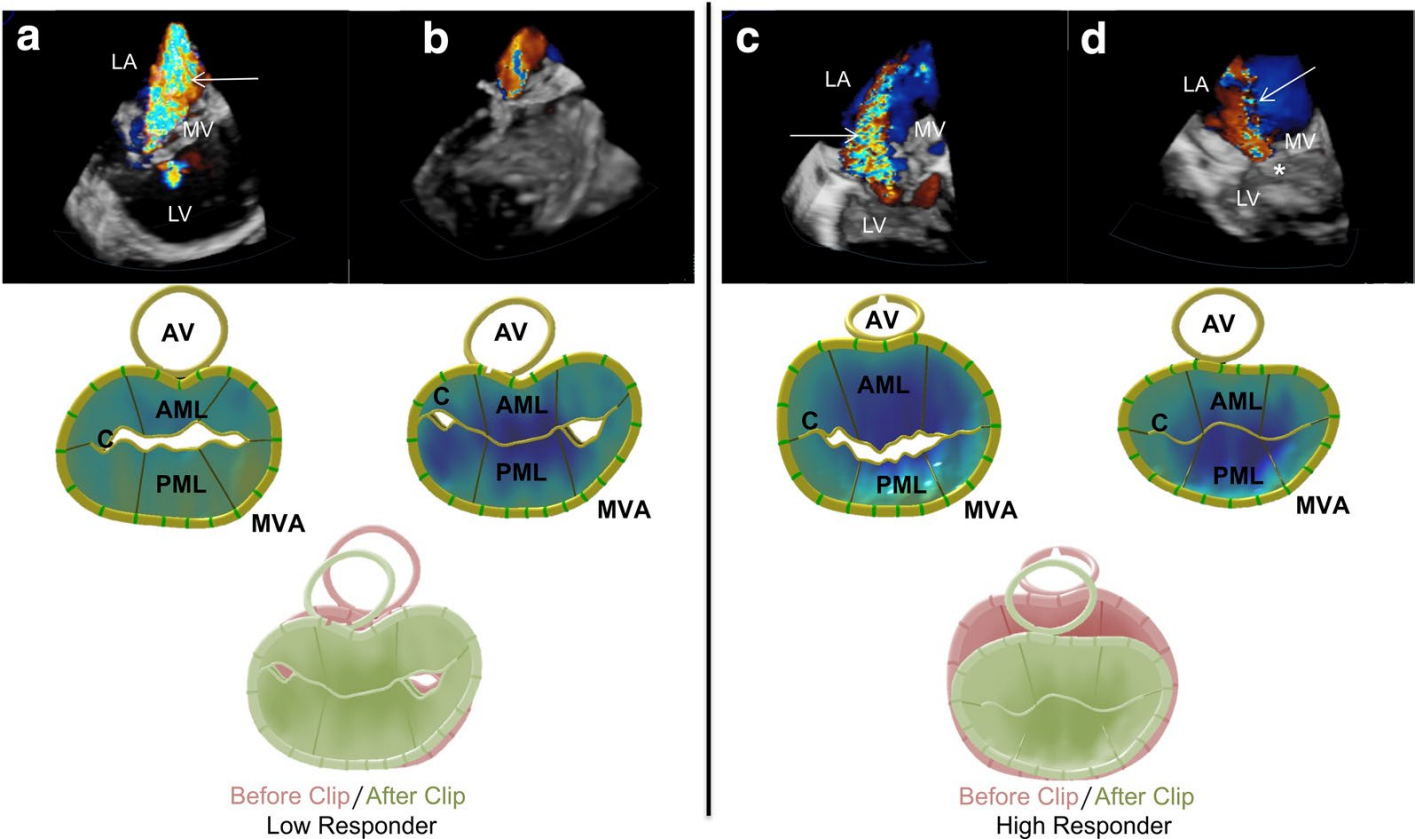
Exemplary 3D models of the acute effects of TMVR on the mitral valve of Low Responder (left) and High Responder (right). Upper picture: Overview of three-dimensional transesophageal echocardiography images comparing Low and High Responder before and after TMVR. Mitral regurgitation jet is visible (right arrow) as well as the MitraClip^®^ device (asterisk) itself. **a** 3D TEE image of Low Responder before TMVR showing severe MR. **b** 3D TEE image of Low Responder after TMVR showing mild MR. **c** 3D TEE image of High Responder before TMVR showing severe MR. **d** 3D TEE image of High Responder after TMVR showing mild MR. Lower picture: Left: 3D model of mitral valves (MV) before and after TMVR of Low Responders with corresponding colored models placed on top of each other to demonstrate change in annular size. Model of MV before and after clip with little change in annular size. Right: Model of Qlab MV before and after TMVR. Change in annular size is more prominent here. *AML* anterior mitral leaflet, *AV* aortic valve, *C* circumference, *LA* left atrium, *LV* left ventricle, *MV* mitral valve, *MVA* mitral valve annulus, *PML* posterior mitral leaflet, *target* indicates mitral regurgitation jet, asterisk indicates clip

The diversification of mitral regurgitation based on (patho-) anatomical and (patho-) physiological aspects in degenerative (DMR) and functional mitral regurgitation (FMR) has tremendous implications for transcatheter interventions [19, 20]. In the EVEREST II cohort, 73% of all patients suffered from structural heart disease with DMR and less than 27% were heart failure patients with FMR [21]. Successful MitraClip^®^ implantation in a high-risk cohort consisting of heart failure patients with FMR [22] was first described in 2010. It could be demonstrated that in patients with FMR, not in those with DMR, MitraClip^®^ therapy leads to significant reduction in mitral valve orifice area during the procedure. There is only one other study demonstrating the correlation between changes in mitral valve anatomy during the procedure and clinical outcome [10]. In that study, an acute reduction of the 3D annulus area and the 3D sphericity index in 107 patients with FMR could be demonstrated. The acute reduction of the 2D DAP was associated with improved clinical response after 6 months. Our results are in accordance with the results of that study regarding the fact that changes in MV geometry occur only in patients with FMR. In addition to that, we could demonstrate significant changes in original 3D data (C3D). The new aspect we gained from our data is that reverse remodeling of the mitral annulus in heart failure patients with FMR only occurred in patients with great clinical improvement at 6-month follow-up, indicating a stronger influence on clinical outcome. Figure 4 impressively indicates an example of the changes of the mitral annulus in the High Responder group, while mitral regurgitation severity is equally reduced in both groups (also compare Fig. 2). A different study with a similar patient population showed the acute effects of TMVR on the mitral valve leaflets demonstrating that through clipping the anterior and posterior leaflet, leaflet stress is not increased [23]. However, the study could not show significantly reduced 3D parameters as shown in this study as well as previous ones [9, 10].

Under the premise that greater reduction in 3D mitral annular geometry yields greater clinical benefits for the patients, the goal of the intervention may shift from mere MR reduction to focusing the procedural goal to reduction of mitral annular geometry using 3D TEE.

### Delineation of mitral annulus remodeling using 3D TEE

As recommended in current guidelines, mitral valve disease is best characterized using 3D TEE [16, 24]. The complex relation between annulus, the saddle shaped ring, and the leaflets can only be described adequately using this technique, especially when it comes to the preparation for MitraClip^®^ interventions [25]. In our study, we used dedicated software for the constitution of a 3D model of the mitral valve apparatus that can very precisely detect minimal changes in mitral ring plasticity during the procedure (Fig. 4). Although these analyses were performed offline, it is possible to perform them during the procedure in order to give the interventionist additional information on the current geometry of the mitral annulus under influence of one, two, or even more clips, irrespective of the interventional result concerning the color flow mapping of regurgitation severity. Our results underline the importance of 3D TEE for diagnosis, preparation, and monitoring the mitral valve apparatus during MitraClip^®^ procedures as only this technique is capable to adequately describe this complex structure, as already described [26]. In our Qlab results (cf. Table 3), the 2D circumference of the mitral annulus was reduced in both groups, while the 3D circumference was only reduced in the High Responder group. These results demonstrate that only the combination of the reduction of the circumference in the dimensions is associated with high response to the MitraClip^®^ procedure. In a recent study, it was demonstrated that 3D changes of the mitral annulus only occur in patients with FMR immediately after Clip implantation. The team used a comparable software as has been used in this study in order to demonstrate a reduction in 3D annulus area, anterior-to-posterior diameter, and 3D tenting area [9]. In contrast to our study, they could not demonstrate a clinical benefit of these changes for patients with FMR. Most importantly, they could not demonstrate any difference in original 3D measurements of mitral annular circumference.

### Clinical response to TMVR in heart failure patients with FMR

Our results demonstrate that the additional effect of TMVR leading to reverse remodeling of the mitral annulus leads to improved clinical outcome than reduction of MR severity alone. Besides the impressive improvement in New York Heart Association functional class, initial NYHA class was higher in patients with high response to the MitraClip^®^ procedure. This underlines our hypothesis that subjects with FMR and heart failure who suffer from severe NYHA class improve after successful mitral annulus remodeling. In contrast to other studies, we could not document any other changes of the cardiac chambers or left ventricular function that correlated with an improved clinical response to TMVR. Recently, it could be demonstrated that changes of left ventricular volumes are associated with improved ejection fraction in heart failure patients with FMR. These changes were associated with a decrease in NT pro-BNP and an increase in the 6-min walk distance [7]. In our population, no significant changes of heart chamber dimensions or ejection fraction could be detected. This might me due to the fact that we included patients with very low ejection fractions compared to other studies. Although the remodeling process seems to start with changes in mitral annulus area directly during the procedure as demonstrated by our results, changes in LV dimensions and LV function therefore might occur later in our population. In both groups of this study, left atrial area showed to be mild to moderately dilated and was not affected by the procedure. Previous studies have shown that dimensional changes in left atrial area do not occur as early as at 6 months after the procedure; however, left atrial global contractile function improves as early as at 3 months after the procedure [27]. Therefore, continuous investigation over a longer period of time (>6 months) may help analyze the left atrial area more precisely and help us understand this process. A different group could demonstrate that the amount of left heart chamber remodeling including the left atrium and the left ventricle depended on the magnitude of MR reduction [8]. We cannot reproduce these results, most likely due to the fact that we only included heart failure patients with FMR, while that group included both—patients with DMR and FMR with better LV function. Also, there were significantly more patients with negative left atrial remodeling and atrial fibrillation in the Low Responder group at baseline. The improved outcome of our High Responder group might partially be due to the fact that there were fewer cases of atrial fibrillation, and the remodeling of the mitral annulus in these patients might have a maintaining effect upon a regular sinus rhythm for improved life quality. This is underlined by the fact that in our High Responder group we had a significant reduction in BNP after 6 months, as already demonstrated by others [7]. In another study addressing this issue, an additional benefit of TMVR on right ventricular function and pulmonary artery pressure in patients with FMR [28] could be shown. We could also demonstrate an effect upon pulmonary artery pressure only in the High Responder group, but there were no changes in right ventricular dimensions and function in our study collective. This also might be due to the fact that these changes might occur later in our population consisting of end-stage heart failure patients.

## Conclusion and future perspectives

In heart failure patients with FMR, TMVR with acute reverse remodeling of the mitral annulus has a tremendous impact upon clinical outcome. Downsizing the mitral annulus seems to be more important than reduction of MR severity alone. This understanding will assist to evaluate the benefit of the procedure while performing TMVR. Furthermore, although the quality of 2D techniques with color Doppler is already very distinguished, 3D TEE has not yet gained enough acceptance among most interventional cardiologist. Considering the great evaluating benefit of 3D models of the mitral valve apparatus as have been used in this study, our results may help to overcome this reluctance.

This will be even more of importance during the evaluation of techniques that in contrast to the edge-to-edge technique address the remodeling of the mitral annulus alone. Recent data demonstrate that indirect annuloplasty devices such as the Carillon^®^ device achieve reduction of mitral annulus dilation and reduction of MR via the coronary sinus [11]. The Titan II trial demonstrated that in patients with FMR, heart failure symptoms improved significantly after reducing the size of the mitral annulus using this approach. Our observations additionally support the assumption that in patients with FMR, reverse remodeling of the annulus plays a major role in the outcome of these patients. Further research is necessary to evaluate which technique is more effective in reducing FMR: edge-to-edge, annuloplasty, or the combination of both by means of interventional hybrid techniques [29].

## Limitations

The number of patients in this study is relatively low due to the fact that this study has a single-center character. Further investigations using a multi-center study approach, including a higher number of patients, might be helpful in order to emphasize our results. Also, we did not show that the remodeling of the mitral annulus alone without reduction of mitral regurgitation has similar or even greater effects. For that, a prospective, randomized, two-group study needs to be performed. An experienced echocardiographer performed all echocardiographic images and another single, well-trained investigator performed all anatomical measurements. However, the data have not been validated independently. Statistically non-significant findings of C3D and DAP in Non-Responders may be due to the sample size and statistical power. A multivariate analysis to define echocardiographic parameters as independent predictors was not used in this study.

## Abbreviations

2D: two-dimensional
3D: three-dimensional
C2D: 2D circumference
C3D: 3D circumference
DAlPm: anterolateral to posteromedial diameter
DAP: anterior to posterior
DMR: degenerative mitral regurgitation
FMR: functional mitral regurgitation
HR: high responders
LR: low responders
LV: left ventricular
MR: mitral regurgitation
NT pro-BNP: N-terminal pro b-type natriuretic peptide
NYHA: New York Heart Association
PAsys: systolic pulmonary artery pressure
TEE: transesophageal echocardiography
TMVR: transcatheter mitral valve repair
TTE: transthoracic echocardiography
VC: vena contracta
